# Pass Rates of Return to Sport Test Batteries Following Anterior Cruciate Ligament Reconstruction: A Systematic Review and Meta-Analysis

**DOI:** 10.3390/sports14050211

**Published:** 2026-05-20

**Authors:** Dominic Richmond, Caroline White, Thomas Gomulko

**Affiliations:** 1 School of Sport, Exercise and Rehabilitation Sciences, University of Birmingham, Edgbaston, Birmingham B15 2TT, UK; 2 Physiotherapy Department, Cambridge University Hospitals NHS Foundation Trust, Addenbrooke’s Hospital, Cambridge CB2 0QQ, UK; 3 Faculty of Health and Human Sciences, School of Sport and Health Sciences, University of Lancashire, Preston, Lancashire PR1 2HE, UK

**Keywords:** anterior cruciate ligament, return to sport testing, anterior cruciate ligament reconstruction, return to sport tests

## Abstract

The association between return-to-sport test batteries (RTS-TBs) and clinical outcomes remains unclear. Previous systematic reviews of RTS-TBs have reported low pass rates; however, these reviews have been limited by substantial heterogeneity. This systematic review aimed to quantify RTS-TB pass rates and examine their association with timing (post-op). Five electronic databases (AMED, CINAHL, MEDLINE, SPORTDiscus, PubMed) were searched on 22 December 2024. Observational studies reporting RTS-TB outcomes as a single pass or fail were included. A random-effects proportion meta-analysis was used to estimate the pooled prevalence of pass rates. A meta-regression was performed to assess the association between test timing and pass rate. Twelve studies (n = 1977) met the eligibility criteria, but five were excluded from the meta-analysis and meta-regression due to overlapping cohorts. From the remaining eight studies (n = 1449), the pooled prevalence of pass rates was 33% overall (95% CI 19 to 47%), 26% (95% CI 18 to 33%) for non-professional athletes, and 73% (95% CI 66 to 80%) for professional athletes, although only a single study focused on professional athletes. No association was observed between the post-operative timing of the test and passing RTS-TB (*p* = 0.73). The observed RTS-TB pass rates are low, and this may be influenced by the extreme heterogeneity. Although no association was observed between the RTS-TB timing and pass rates, this finding alone cannot confirm causality.

## 1. Introduction

Anterior cruciate ligament (ACL) injuries are a growing public health and economic concern in the United Kingdom, with approximately 30,000 injuries per annum [1,2]. ACL injuries can be managed with rehabilitation alone [3] or with ACL reconstruction (ACLR) surgery followed by postoperative rehabilitation [4]. ACLR rates increased twelvefold between 1997 and 2017 in England and Wales [1], resulting in an estimated annual cost to the NHS of £65 million [2]. This suggests that ACLR is the overwhelming treatment preference [5]; as such, high-quality rehabilitation is important if rising surgical volumes are to yield proportionate improvements in patient outcomes.

The increasing demand for surgery is met with similarly high patient expectations. For instance, just 60% of non-professional athletes have returned to their preinjury sport level at two years post-surgery [6]. Yet, 88% of individuals expect to return to their preinjury level of sport within the first year of ACLR [7], which is more comparable with professional athletes [8]. Furthermore, those who are unable to return to sport frequently report a reduced quality of life [9], and 25–39% experience a reinjury [10]. The gap between expectation and outcome raises questions about the factors influencing these outcomes. Consequently, establishing confidence in the rehabilitation process is a necessary step in answering these questions.

Return to sport test batteries (RTS-TB) combining time, strength, functional, and patient-reported outcome measures (PROMs) ascended into clinical practice following the suggestion that reinjury risk could be reduced by up to 84% [11,12]. However, the overall findings are equivocal, with evidence demonstrating little prognostic utility or even negative associations, which undermines confidence in their predictive validity [13]. The inconsistency of results reflects the methodological variability of RTS-TBs within these studies; yet, individual tests, such as strength, hop tests, and PROMs, demonstrate good reliability [14,15,16]. Therefore, as practice has shifted towards criterion-based rehabilitation, it is worth considering that the benefit of RTS-TB may lie less in predicting outcomes and more in affirming the quality and completeness of rehabilitation itself.

Previous reviews have demonstrated RTS-TB pooled pass rates of 23–44% [17,18]; however, both reviews had influential limitations. Hurley et al. [19] limited the meta-analysis to studies reporting reinjury rates, while Webster and Hewett [18] included a large time span with a lenient inclusion criterion; both studies included studies with outdated rehabilitation. Combined, there is a concern that these results are diluted, thereby misrepresenting accurate pass rates of modern rehabilitation. Therefore, this systematic review aims to re-evaluate the pooled prevalence of pass rates of RTS-TB following ACLR and examine whether the timing of testing influences the likelihood of passing. It was outside the scope of this review to evaluate the predictive value of RTS-TB on reinjury or RTS success.

## 2. Materials and Methods

This systematic review, including the meta-analysis and meta-regression, was PROSPERO registered (CRD420251120766). A study protocol and search strategy were published and are available online (see Supplementary Materials). This review was conducted in line with the Preferred Reporting Items for Systematic Reviews and Meta-analysis (PRISMA) statement [20], and PRISMA in Exercise, Rehabilitation, Sport medicine and SporTs science (PERSiST) guidance [21]. Covidence systematic review software (Covidence, Veritas Health Innovation, Melbourne, Australia) www.covidence.org (accessed on 22 December 2024) was used for the management of the search strategy and studies yielded.

### 2.1. Search Strategy

One author (DR) created the search terms and searched five electronic databases (AMED, CINAHL, Medline, SPORTDiscus and PubMed) from 1 January 2016 to 22 December 2024. Search terms derived from a PICO table were developed for the EBSCO search tool and were adapted for PubMed. Terms from each keyword concept were combined with the ‘AND’ Boolean operator, while concepts were combined with the substitute terms using the ‘OR’ Boolean operator. Each keyword term was restricted to ‘title’ or ‘abstract’. Citations of the included studies were checked for any unidentified studies. The full search strategy is provided in the online supplemental file (see Supplementary Materials).

### 2.2. Study Selection

A systematic literature search was conducted on 22 December 2024, with the date range set from 1 January 2016 to 22 December 2024, to identify studies for inclusion. The search limits were set to only include peer-reviewed journals and excluded reviews and books. Two reviewers conducted the initial search. Once full-text screening was completed, a secondary search was performed through the citations of eligible articles. Both researchers had to agree on the relevance of any included studies. The date range was set from 1 January 2016 to 22 December 2024, due to the impact of four best practice guidelines released in quick succession on the management of ACL-R and RTS publications in 2015 and 2016 [22,23,24,25]. These publications likely had an influence on the rehabilitation of ACL due to the suggestion of extensive use of RTS-TB and a focus on criterion-based milestones. Therefore, the aim of this review was to capture publications produced since then to investigate the influence they may have had on subsequent rehabilitation and clinical practice.

Studies were included if: (1) they were observational studies; this design format allows for observation of participants in a real-world context and allowed for larger data pools than RCTs, thus enhancing external validity; (2) participants had undergone ACLR; conservative management, cross-bracing and ACL repairs were not included due to differences in care pathways that would introduce additional clinical heterogeneity; (3) participants performed a RTS-TB: defined as a cluster of tests which included quadriceps testing and a hop test, at the minimum, consistent with the clinical guidelines; (4) RTS-TB was reported as a single outcome, ‘pass’ or ‘fail’; a set of pre-determined tests with a specific cut-off (e.g., ≥90% LSI on all tests) resembles the best practice guidelines; (5) the full text was available in the English language; we had insufficient resources to translate non-English texts.

Studies were excluded if: (1) They contained >2 PROMs contributing to the test criteria; inclusion of PROMs reflects the multidimensional framework of rehabilitation. However, it was crucial not to disproportionately weight them and risk skewing results towards PROMs rather than objective performance. The IKDC and ACL-RSI are two frequently cited PROMs with established prognostic validity. The threshold was capped at two to ensure that things remained clinically applicable and the PROMs complemented the RTS-TB, rather than dominated it. (2) They contained ≥10 separate items in the test criteria; a critique of the previous reviews is the possibility that excessive test numbers increase the likelihood of failing RTS-TBs. Introducing a threshold of nine tests was prespecified in the PROSPERO protocol; this was selected to allow for a comprehensive RTS-TB with sufficient coverage of hop tests, strength tests, PROMs and functional tests but without excessive testing burden. (3) Studies were based on an exclusively paediatric population (at or below 16 years old); mixed age brackets are applicable to clinical services that deliver ACLR care. However, there are specific clinical considerations for paediatric patients that would make results less generalisable to the adult population. (4) They were single case studies; these were excluded as they represent the lowest level of clinical evidence.

### 2.3. Data Extraction

Two authors completed data extraction independently using the Covidence template. For each study, data on country of origin, sex, age, test composition, time of testing, pass criteria and pass rate was collected. When insufficient data were published, authors were contacted to provide missing data where possible.

### 2.4. Risk of Bias Certainty of Evidence

Two reviewers independently appraised each study using the Joanna Briggs Institute (JBI) critical appraisal tools for prevalence studies [26]. The JBI tool does not produce a risk of bias (RoB) score; therefore, a three-tier classification of low, moderate or high was used for each study, analogous to the Newcastle–Ottawa Scale [27], to demonstrate the authors’ opinion on RoB. Studies scoring ≤ 3 ‘yes’ were high risk of bias, 4–6 were moderate risk of bias and 7–9 were low risk of bias.

### 2.5. Statistical Analysis

A narrative synthesis was used to analyse any data which was not used in a meta-analysis, using the synthesis without meta-analysis (SWiM) guidelines [28]. The primary outcome was the RTS-TB pass rate (percentage), and the secondary outcome was time of testing (months).

A random-effects meta-analysis of proportions was performed in Stata 18.5/SE (StataCorp. 2025, College Station, TX, USA). The pooled proportions were estimated using the Freeman–Tukey double arcsine transformation to stabilise variances and accommodate extreme proportions, including zero events, without requiring continuity corrections [29]. A sensitivity analysis was conducted using logit-transformed proportions for robustness, including a subgroup analysis of non-professional groups, and a leave-one-out analysis was performed. A meta-regression was performed with a logit transformation to assess the association between the timing of tests and pass rates. Logit-transformed proportions were calculated using a continuity correction of 0.5, which is consistent with recommendations for handling zero cells in meta-analysis of proportions [29]. Where more than one time point was provided, the latest was used to capture the entirety of the rehabilitation process.

### 2.6. Equity, Diversity, and Inclusion

Participants in the review were from a variety of geographic locations, including Europe, Australia, North America and the Middle East. However, more than 50% were based in North America. This may limit the generalisability of results to other parts of the globe. Finally, this study aimed to include participants with ACL injuries regardless of sex, socioeconomic level, country of origin or level of sport.

The research team (3) comprised personnel from a mixture of academic backgrounds and career stages (student, lecturer and senior lecturer); and a mixture of clinical expertise (specialist sports physiotherapist, advanced practice physiotherapist in knees and specialist amputee physiotherapist). Furthermore, the research project included underrepresented groups within academia, including members from BAME (1) and female backgrounds (1).

## 3. Results

**Figure 1:**
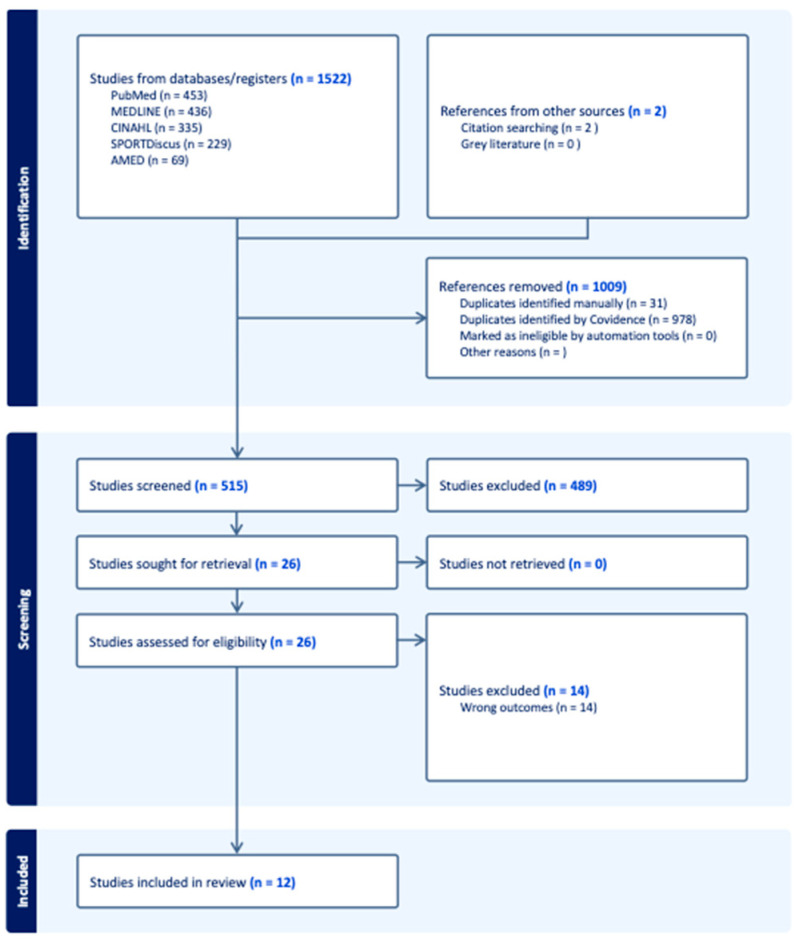
PRISMA flow diagram of study selection. Blue bold text refers to the total number of studies included (left) or excluded (right).

The database search yielded 1522 articles, and two articles were found through citation searching. Duplicate removal occurred in two stages, with the first being the use of the automated Covidence tool. The second was a manual removal during the abstract and title screening. Five hundred and fifteen articles remained after the removal of duplicates. Twenty-six studies remained for full-text review after the title and abstract screening. Following full-text review, 12 studies were included in the systematic review for data extraction and analysis (see Figure 1), with a total of 1449 participants.

Ten of the 12 studies were reported to have moderate RoB; meanwhile, one was low RoB [11] and one study demonstrated a high RoB (see Table 1) [30]. The study characteristics are listed in Table 2. Three research cohorts were reported on in numerous publications, which resulted in overlapping participant cohorts (ACLRELAY [31,32,33], Delaware–Oslo [11,30,34] and Swedish ACL registry [35,36]). Therefore, of those eligible for the meta-analysis and meta-regression, the study with the largest cohort from each group was used in the meta-analysis, and the remaining cohorts were excluded to minimise the selection and sample bias.

### 3.1. Meta-Analysis and Meta-Regression

**Figure 2:**
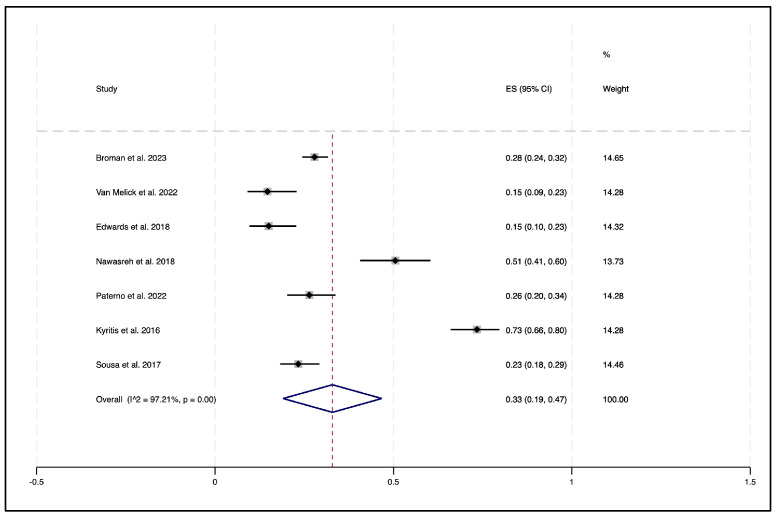
Forest plot displaying the overall proportion of athletes passing the return-to-sport test battery (RTS-TB) for eligible studies [5,30,33,36,37,39,40]. Each square represents the proportion from an individual study, with the size of the square proportional to the study’s weight in the meta-analysis. Horizontal lines represent 95% confidence intervals. The diamond represents the overall pooled proportion, with its width corresponding to the 95% confidence interval. The dashed vertical line indicates the pooled estimate. Heterogeneity was assessed using the I^2^ statistic (I^2^ = 97.21%, *p* = 0.00), indicating substantial heterogeneity between studies.

**Figure 3:**
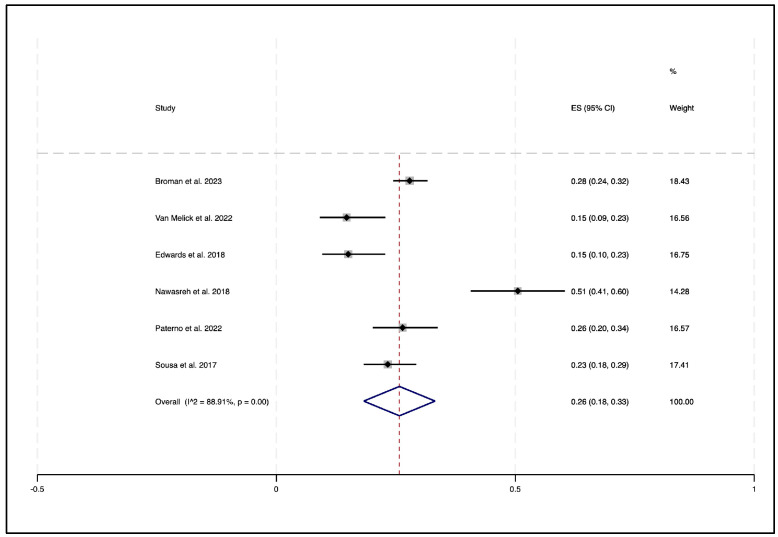
Forest plot displaying the sub-group of non-professional athletes passing the return-to-sport test battery (RTS-TB) for eligible studies [30,33,36,37,39,40].

The random-effects meta-analysis using the Freeman–Tukey transformation showed that 33% (95% CI 19–47%) of participants passed an RTS-TB following ACLR (see Figure 2). A sensitivity analysis using logit-transformed proportions demonstrated a comparable estimate of 31% (95% CI 19–46%). A subgroup analysis of the non-professional athletes revealed a lower pooled pass rate of 26% (95% CI 18–33%) (see Figure 3). By contrast, the single study involving professional athletes had a substantially higher pass rate of 73% (95% CI 66–80%).

Statistically, there was evidence of differences between studies (Chi^2^ = 215.34, df = 6, *p* < 0.001), indicating extremely high heterogeneity (I^2^ = 97.21%, Tau^2^ = 0.03). Heterogeneity was reduced when the single professional athlete study was excluded (Chi^2^ = 45.10, df = 5, *p* < 0.001; I^2^ = 88.91%, Tau^2^ = 0.01), though it remained high.

**Figure 4:**
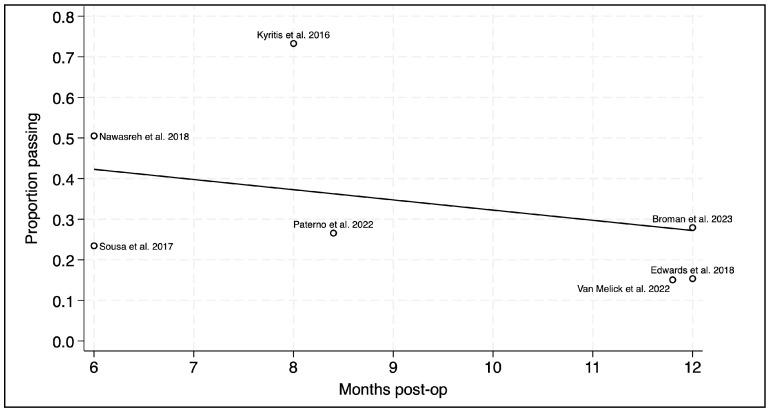
Meta-regression. The association between timing of the test (post-op) and RTS-TB pass rates [5,30,33,36,37,39,40].

Meta-regression analysis did not demonstrate a significant association between the timing of the RTS test (see Figure 4) and the proportion passing rate of the RTS-TBs (*p* = 0.211 with all studies; *p* = 0.150 without Kyritsis et al. [12]. Additional meta-regressions exploring separate study variables such as graft choice, age, gender, geographical location, test composition and criteria definition were not explored quantitatively due to insufficient availability of data, likely leading to inadequate power of the statistical tests; therefore, these variables will be highlighted in the narrative synthesis and interpreted in the discussion.

### 3.2. Narrative Synthesis

Patient demographics varied across the studies included. For instance, there were differences in the age ranges across several studies, with some demonstrating a narrow age range from early adolescence to early adulthood [32,33,41] and others a quite broad one [11,30,34,38,40]. Just one study used an adult exclusive cohort [35], and three did not report the age range [5,31,37], though they did report mean values.

Additionally, there were variations in participant sex (expressed as % female) across studies. Just one study [5] included an exclusively male cohort, while the remainder of the studies had a mixed gender cohort.

Of the included studies, six were undertaken in North America (USA), four in Europe (Norway, Netherlands and Sweden), one in Australasia (Australia) and one in the Middle East (Qatar). Finally, there was high variation and inconsistent reporting of the participants’ level of sport; therefore, comparable data could not be extracted across studies.

The timing of RTS-TB ranged from 6 to 12 months, with three studies opting for the earlier 6 months [30,34,40]. However, the remaining studies ranged from 7 to 12.3 months.

Variation was observed between quadriceps strength testing protocols, with just three studies sharing a knee extensor strength protocol (single test, 90 º/s isokinetic test) [30,35,37]. The remaining studies used a variety of testing modalities, contraction types and speeds. Similarly, hamstring testing was used with inconsistent speeds and contraction types; however, just 7 of 12 studies included hamstring testing [5,31,35,37,38,40,41], despite its biomechanical relevance as an agonist to the ACL.

Six hop tests were identified, and these were used in four separate clusters. Test clusters could be divided into Gustavssons et al. [15] and Noyes et al. [14] and two clusters which had not previously been published [5,40]. There was a trend of the USA-based studies using Noyes et al. [14] and the European studies using the Gustavsson et al. [15] protocol (see Table 2).

### 3.3. GRADE

The level of certainty started as low for each phenomenon, due to the use of observational studies [29] (see Table 3). The evidence was further downgraded for RoB, inconsistency (heterogeneity between study protocols and populations), indirectness (non-UK settings, variable RTS-TB methods, lack of Tegner activity scale stratification) and imprecision (wide confidence intervals and a single study subgroup analysis). Publication bias was not formally assessed using statistical tests, as these methods would likely be unreliable with so few studies. However, where publication bias could not be excluded, it was downgraded. Overall, the certainty was very low for the pooled pass rates of the overall meta-analysis, subgroup and association between timing and pass rates. Consequently, the results of this review will be followed by research and clinical implications, rather than recommendations.

## 4. Discussion

This review aimed to evaluate the pooled RTS-TB pass rates following ACLR and the association between the time of testing and pass rates. It was the first review to examine the association between test timing and pass rates and focus on the rehabilitation studies published following the guidelines released in 2015–2016 [22,23,24,25]. The overall pass rate was 33%, with substantial heterogeneity (I^2^ = 97%, Tau^2^ = 0.03) across participant characteristics and protocols. This is higher than the previously reported 23% [18] but lower than 44% [19]. The pass rate reduced to 26% in non-professional athletes, similar to Webster and Hewett [18]. A higher pass rate (73%) was observed in a single study on professional athletes; however, it lacks the statistical power to draw firm conclusions. Nonetheless, it may carry important implications for understanding the determinants of RTS-TB. This warrants further investigation and will be explored speculatively through this discussion.

This review aimed to use a more stringent inclusion criterion; however, the methodological variation remained high (I^2^ = 97%, Tau^2^ = 0.03), similarly to previous reviews (95% CI 8–43%, I^2^ = 97.5). The extremely high heterogeneity observed substantially limits the clinical interpretability of the pooled prevalence estimate. Although the RTS-TB pass rates provide a useful overall signal that relatively few individuals pass following ACLR, it should not be applied as a benchmark. Instead, it reflects an average across studies where there are major differences. While the random effects model accounts for this variation statistically, it does not resolve the inconsistencies seen across research and clinical practice. Therefore, the pooled estimate is most applicable as a broad indicator of RTS-TB successful outcomes, rather than a precise target.

### 4.1. Participant Characteristics

Patient characteristics, such as age, are important because of the incidence of concomitance and reinjury in paediatric and adolescent populations [10,39]. Younger individuals benefit from more supervision with rehabilitation [42], early intervention [43] and sometimes require more rigorous procedures, such as LET (lateral extra-articular tenodesis) [44,45]. Therefore, the pooling of different age groups introduces age as a confounding variable, which introduces selection bias and reduces the generalisability of these results.

Participant sex was reported in all studies, and all but one study were mixed male and female cohorts; however, there was one male-only study [5]. Furthermore, there were three studies where those who were eligible for testing differed from the original cohort, and these studies failed to report the sex distribution at the time of testing. Due to the importance of age, sex and sport participation level in ACLR research, it is essential that future outcomes on RTS-TB and RTS are stratified accordingly.

### 4.2. RTS-TB Configuration

The relationship observed between time and prognostic factors, such as reduced reinjury rates in previous studies, made the investigation of time and RTS-TB pass rates worthwhile [11,36,46]. The timing of testing ranged from 6 to 12 months, which is a similar range to previous reviews [18]. This review observed no association between RTS-TB timing and pass rates within that six-month window; however, the narrow margins examined may have limited the variability and statistical power of these results. Prognostic factors were not explored in the current review. Therefore, one can only speculate on the reasons that time may be associated with prognostic factors but not RTS-TB outcomes. For instance, the consideration of the role that time plays in graft maturation is likely to be important for clinical outcomes [47]. In reality, the link between time and graft maturation is complex and involves passive tissue healing/ligamentization [48]. Additionally, increased physical demands on the graft accrued over time may lead to a greater cross-sectional area and tensile load, similar to the native ACL. Meanwhile, the physical performance markers such as range of motion, strength, power and proprioception, which make up RTS-TB, are seen as adaptations that require a physical training stimulus [49,50,51]. Therefore, the lack of association observed between the time of testing and RTS-TB may be due to methodological limitations or the passive role that time has when it is independent from a sufficient training stimulus. As such, clinicians are reminded that the individual readiness of athletes, combined with time, likely supersedes time alone for clinical outcomes.

Central to individual readiness is the concept of a staged criterion-based rehabilitation. Staged criterion refers to the sequential and progressive implementation of milestones in rehabilitation [52,53,54,55,56,57,58,59]. This has become a cornerstone of ACLR rehabilitation practice over the past two decades [11,52,53,54,55,56,57,58]. Interestingly, the studies in this review applied the RTS-TB as a single time-point decision for a return to sport, or clinical discharge. Furthermore, some studies let the participants return to sport before testing, which introduces performance bias and risk to the patient. Consequently, whether earlier milestones were achieved, such as full knee extension [60], overcoming arthrogenic muscle inhibition, and swelling management, remains unclear. It is plausible that the inadequate progression through earlier stages may limit one’s ability to pass an RTS-TB due to the disrupted review and action feedback loop required for clinicians to adapt rehabilitation [61]. Therefore, the use of a single-stage RTS-TB approach has methodological limitations; additionally, it limits the transparency of the rehabilitation process, making it difficult to examine whether variables in rehabilitation influence the RTS-TB pass rates.

### 4.3. RTS-TB Content

There was significant variation in the content of RTS-TBs, with no studies sharing an RTS-TB protocol. For instance, hamstring strength testing was absent in 5 of 12 studies. The association between hamstrings and the quadriceps to hamstrings (Q/H) and reduced risk of reinjury [5] has been repeatedly refuted by high quality studies [62,63,64]. However, the importance of hamstring strength may relate to the frequent use of hamstring grafts and its agonist role in offloading anterior tibial translation force at the ACL [65,66]. This likely contributes somewhat to the influence that neuromuscular injury prevention programmes have on reduced injury prevalence in female athletes [67]. Including hamstring testing is unlikely to increase RTS-TB, but the omission does increase the inconsistency and indirectness of the results, which lowers the GRADE assessment.

On the other hand, quadricep strength testing was featured in all studies, but varied in the number of tests performed, contraction type, and contraction speed. Quadricep strength has been associated with increased confidence in RTS, RTS outcomes, and reinjury risk [68]. Therefore, the validity of hand-held (ICC = 0.70, 95% CI 0.54 to 0.82) [69] and in-line dynamometry testing (ICC = 0.97, 95% CI 0.94 to 0.98) is valuable to clinicians because of increased feasibility [70]. However, isokinetic dynamometry is the gold standard for quadricep strength testing [69,70], and this review highlights the inconsistency of testing methods across studies. Furthermore, emerging evidence suggests that eccentric quadricep strength may be fundamental, due to the resemblance it bears to common mechanisms of injury [71,72,73]; therefore, we question the construct validity of concentric and isometric contractions. Therefore, despite many reliable methods of quadricep strength testing, this study highlighted that research and clinical practice lack consistency, which prevents important comparative data.

Functional tests and PROMs were seldom used across studies; however, hop tests were used across all studies. Just two of the four clusters had publications demonstrating strong reliability [14,15]. However, Cooke et al.’s [74] synthesis of lower limb functional tests highlighted that while all of the tests featured in this review demonstrate strong reliability, some did not demonstrate adequate validity. For instance, the triple hop, timed 6 m hop, and triple crossover are not validated tests for injury prevention, and their biomechanical relevance to knee extensor strength and knee injury prevention has been challenged [74,75]. Nonetheless, hop tests are low-cost alternatives that can provide reliable information in the absence of equipment that accurately records biomechanical data. Although there is yet to be validation of hop tests, their reliability makes them a low-cost tool that is likely to be beneficial for clinical practice and observational studies, but this review highlights the need for further standardisation.

### 4.4. Professional vs. Non-Professional Athletes

There was a substantially higher RTS-TB pass rate among professional compared to non-professional athletes (95% CI, 73% vs. 26%, respectively). If meaningful, this mirrors the disparity between the return to the preinjury sport level observed between professional (85.8%) [8] and non-professional athletes (43–60%) [6,76]. D’Ambrosi et al.’s [8] review provides a good level of statistical power to this assertion with the large number of participants included (n = 4463, 95% CI 82.8–88.5%), whereas this review lacks a similar statistical power, with just a single study providing data on professional athletes. Nonetheless, further research is required to explore whether there is an impact from factors such as longer periods of supervised rehabilitation, access to more experienced staff and multidisciplinary teams afforded to professional athletes [77,78]. The precedent for this may be observed in Tabener et al.’s [79] case study, which provides detailed insight into the rehabilitation afforded to several professional athletes. Finally, the suggestion that professional athletes demonstrate increased RTS-TB pass rates is grounded in a single study; therefore, it should serve to generate future research questions on clinical practice and future research on the topic, rather than support the assertion that professional athletes pass at disproportionately higher rates.

### 4.5. Influence of Rehabilitation on RTS-TB Outcomes

A recurrent theme from these findings is the speculation that RTS-TB scores are influenced by possible limitations in the rehabilitation process, rather than RTS-TB limitations. Wood et al. [80] observed that non-injured participants have difficulty passing ACLR RTS-TB, and later implied that RTS-TB may be too challenging. This difficulty is likely related to the number of tests included within an RTS-TB and the mathematical implications that this may have on passing [81,82]. This review observed the duplication of quadricep strength testing and hop testing (triple hop and triple cross-over hop test), but it is unclear whether this contributed to the low pass rates. Furthermore, the studies included in Wood et al.’s [80] review did not use rehabilitation or training with the non-injured group, which makes the results of RTS-TB less comparable with those following an ACLR rehabilitation. This undermines the argument that RTS-TB are too difficult, but it does highlight the need for greater selectivity with test inclusion.

Incomplete rehabilitation is likely to be one limitation in rehabilitation. Receiving less than 6 months of supervised rehabilitation has been associated with scoring < 85% LSI for strength and hopping tests; meanwhile, more than 6 months of rehabilitation was associated with patients scoring ≥ 90% LSI [83]. Additionally, quantitative and qualitative deficits are observed up to 6 months post-ACLR [84]. Therefore, a strong argument exists to suggest that individuals would require at least 6 months of rehabilitation post-ACLR to minimise the risk of reinjury and maximise the possibility of an RTS [37,85]. Ebert et al. [83] observed that only 33% of participants received supervised rehabilitation ≥ 6 months that included structured agility and landing exercises, despite the suggestion that 10 to 12 months seems more appropriate when time and criteria are considered together [86]. Consequently, incomplete rehabilitation poses a plausible limitation to the rehabilitation process, and this could possibly impact RTS-TB pass rates. As such, incomplete rehabilitation provides an alternative explanation rather than high test stringency for the low RTS-TB pass rates.

### 4.6. Limitations

This systematic review had several limitations. For instance, the studies were all vulnerable to confounding variables, which were highlighted in the ROB assessment. Furthermore, despite appearing in a search of the last 8 years, some cohorts included participants from as long as 18–20 years ago [5,11,31,40], which reduces the relevance to current practice. As a collective, much of the heterogeneity between studies seems secondary to the study methodology. Methods of data collection and reporting led to the inability to perform subgroup analysis on the ‘higher risk’ groups, such as adolescents, females, and higher-level activities. Additionally, the overlapping participant pools restricted statistical power by reducing participant pool availability for meta-analysis. Finally, the subgroup analysis on the professional cohort was based on a single study; further comparisons of professional and non-professional athletes will help to improve the certainty of evidence.

### 4.7. Implications

The results of this review carry implications for future research design, clinical practice and patient care. Given the heterogeneity and low certainty of evidence observed in this review, an international Delphi consensus is recommended to standardise the RTS-TB structure, content and reporting standards in this area. Additionally, future research should seek to understand whether improvements to rehabilitation completeness or content increase RTS-TB rates. The United Kingdom requires observational studies on the ACLR population and researchers should proactively seek to stratify results based upon sex, age, graft type and Tegner activity score, which are crucial for the translation of results into clinical practice. Consequently, improvements to the quality of research can be suggested from the findings of this review and they must be actioned before there can be firm conclusions about the association with reinjury risk and RTS-TB outcomes.

There are practical takeaways for clinicians based on the results of this review. For example, clinicians should consider using staged test criteria, rather than the definitive single-time-point tests in this review, because of the low RTS-TB pass rates. Furthermore, in the absence of a standardised RTS-TB, clinicians should do their due diligence and seek to use the tests or milestone with greatest reliability and validity, while being mindful to keep the tests to the minimal amount necessary. This review also demonstrated that time alone is not associated with the likelihood of passing RTS -TBs; thus, clinicians should use this information as part of the ongoing education on the importance of rehabilitation, which may help to address misaligned expectations.

## 5. Conclusions

This systematic review demonstrated an overall RTS-TB pass rate of 33%, with no significant association between the timing of the tests and the likelihood of passing. Notably, there was an almost threefold increase in the pass rate among professional compared to non-professional athletes (73% compared to 26%), although this was based on a single study. The substantial heterogeneity between studies limits the appropriateness of universal recommendations for best practice and subsequent benchmarking. However, the results should prompt a closer examination of rehabilitative practices following an ACLR.

## Figures and Tables

**Table 1:** JBI prevalence checklist.

	1	2	3	4	5	6	7	8	9	RoB
Grindem et al. [11]	Y	Y	Y	Y	N	Y	N	Y	Y	Low
Kyritis et al. [5]	Y	Y	Y	Y	U	U	Y	Y	U	Mod
Sousa et al. [37]	U	Y	Y	Y	U	U	Y	Y	N	Mod
Toole et al. [32]	U	U	Y	Y	N	U	Y	Y	U	Mod
Wellsandt et al. [35]	P	P	P	Y	N	P	Y	Y	N	High
Beischer et al. [38]	Y	Y	Y	Y	P	P	Y	Y	U	Mod
Edwards et al. [39]	U	U	Y	Y	N	Y	Y	Y	N	Mod
Nawasreh et al. [30]	U	U	Y	Y	N	N	Y	Y	U	Mod
Losciale et al. [34]	U	U	Y	Y	U	U	Y	Y	U	Mod
Paterno et al. [33]	U	U	Y	Y	N	N	Y	Y	U	Mod
Van Melick et al. [40]	U	Y	Y	Y	N	N	Y	Y	P	Mod
Broman et al. [36]	Y	Y	Y	Y	U	U	Y	Y	N	Mod

(1) Was the sample frame appropriate to address the target population? (2) Were study participants sampled in an appropriate way? (3) Was the sample size adequate? (4) Were the study subjects and the setting described in detail? (5) Was the data analysis conducted with sufficient coverage of the identified sample? (6) Were valid methods used for the identification of the condition? (7) Was the condition measured in a standard, reliable way for all participants? (8) Was the statistical analysis appropriate? (9) Was the response rate adequate, and if not, was the low response rate managed appropriately?

**Table 2:** Summarised characteristics of the studies included in the present systematic review.

Author (Year)	Participants	Hop Test	Strength Test	Function Test	PROMS	RTS-TB Criteria	Time Post-Op	Results
Grindem et al. [11]	n = 100 (54 Female; 54%) Age 24.3 ± 7.3 (13–60)	Hop for distance Timed 6 m hop Triple hop Triple cross over	Knee extensor: 60º/s	n.a.	KOS-ADLS GRS	≥90% LSI All tests	6.0 months 12.0 months	Pass: 18 (24.7%) Fail: 55 (75.3%)
Kyritsis et al. [5]	n = 158 (0 Female; 0%) Age 21.16 ± 4.18 (n.a)	Hop for distance Triple hop Triple cross over	Knee extensor: 60º/s, 180º/s, 300º/s Knee flexor: 60º/s	Agility *T*-test	n.a.	≥90% LSI T-test < 11 s	8.1 months	Pass:116 (73.4%) Fail: 42 (26.6%)
Sousa et al. [37]	n = 223 (131 Female; 58.7%) Age 26.5 ± 11.8 (12–59)	Hop for distance Vertical hop Triple hop	Knee extensor: 60º/s, 180º/s Knee flexor: 60º/s, 180º/s	n.a.	n.a.	≥90% LSI ≥6/7 tests	6.0 months	Pass: 52 (23.3%) Fail: 171 (76.7%)
Toole et al. [32]	n = 115 (88 Female; 76.6%) Age 17.1 ± 2.5 (n.a.)	Hop for distance Timed 6 m hop Triple hop Triple cross over	Knee extensor: 180º/s Knee flexor: 180º/s	n.a.	IKDC	≥90% LSI All tests	8.0 months	Pass: 16 (13.9%) Fail: 99 (86.1%)
Wellsandt et al. [35]	n = 70 (Female n.a.) Age n.a. ± n.a. (4–55)	Hop for distance Timed 6 m hop Triple hop Triple cross over	Knee extensor: 90º/s	n.a.	n.a.	≥90% LSI All tests	6.0 months	Pass: 40 (57.1%) Fail: 30 (42.9%)
Beischer et al. [38]	n = 100 (Female n.a.) Age n.a. ± n.a. (15–30)	Vertical hop Hop for distance Side hop	Knee extensor: MVIC 60º, 90º/s Knee flexor: MVIC 30º, 90º/s	n.a.	n.a.	≥90% All tests	12.0 months	Pass: 25 (25%) Fail 75 (75%)
Edwards et al. [39]	n = 113 (38 Female; 22.6%) Age 25.9 ± 8.5 (n.a)	Hop for distance Timed 6 m hop Triple hop Triple cross over	Knee extensor: 90º/s Knee Flexor: 90º/s	n.a.	n.a.	≥90% All tests	12.3 months	Pass: 17 (15%) Fail: 96 (85%)
Nawasreh et al. [30]	n = 95 (32 Female; 33.7%) Age 27.6 ± 10.5	Hop for distance Timed 6 m hop Triple hop Triple cross over	Knee extensor: MVIC	n.a.	KOS-ADLS GRS	≥90% All tests	6.0 months	Pass: 48 (50.5%) Fail: 47 (49.5%)
Losciale et al. [34]	n = 148 (100 Female; 67.6%) Age 16.9 ± 3.4 (14–25)	Hop for distance Timed 6 m hop Triple hop Triple cross over	Knee extensor: 60º MVIC	IKDC	n.a.	≥90% All tests	7.0 months	Pass: 39 (26.4%) Fail: 109 (73.6%)
Paterno et al. [33]	n = 159 (112 Female; 70.4%) Age 17.2 ± 2.6 (13–27)	Hop for distance Timed 6 m hop Triple hop Triple cross over	Knee extensor: 60º/s, 90º/s	IKDC	n.a.	≥90% All tests	8.4 months	Pass: 42 (26.4%) Fail: 117 (73.6%)
van Melick et al. [40]	n = 102 (Female n.a.) Age n.a. ± SD n.a (16–50)	Vertical hop Hop for distance Side hop	Knee extensor: MVIC (HHD) Knee flexor: MVIC (HHD) Eccentric Hip abductor: MVIC (HHD)	n.a.	LESS Hop and hold	≥90% LSI strength and hops ‘Yes’ on SL hop and hold OR < 6 on LESS	11.8 months	Pass: 15 (14.7%) Fail: 87 (85.3%)
Broman et al. [36]	n = 588 (303 Female;51.5%) Age 29.3 ± 9.8 (18–65)	Vertical hop Hop for distance Side hop	Knee extensor: 90º/s Knee Flexor: 90º/s	n.a.	n.a.	≥90% All tests	12.0 months	Pass 165 (27.9%) Fail 424 (72.1%)

n.a. refers to not applicable and is used where the study did not include the corresponding test domain.

**Table 3:** Certainty of evidence (GRADE) summary table.

Phenomena of Interest	Participants	Level of Certainty	Reasons for Mark Down
33% participants passed overall	1449	Very low ⊕◯◯◯	1, 2, 3, 4, 5
26% non-professional athletes passed	1291	Very low ⊕◯◯◯	1, 2, 3, 4, 5
73.4% of professional athletes passed	158	Very low ⊕◯◯◯	1, 2, 3, 4

Level of certainty is represented by four circles: each ⊕ (filled circle) denotes a level of certainty met, while each ◯ (open circle) denotes a level not met. The scale ranges as follows: very low ⊕◯◯◯, low ⊕⊕◯◯ moderate ⊕⊕⊕◯ and high⊕⊕⊕⊕. Criteria for marking down: (1) risk of bias; (2) consistency; (3) imprecision; (4) indirectness; and (5) publication bias.

## Data Availability

The original contributions presented in this study are included in the article/Supplementary Material. Further inquiries can be directed to the corresponding author.

## Supplementary Materials

The following supporting information can be downloaded at: https://www.mdpi.com/article/10.3390/sports14050211/s1, S1: Full electronic search strategies. S2: Table of data extraction. S3: Full risk of bias assessments. S4: Statistical analysis. S5: PROSPERO protocol Record. And PRISMA Checklist.

## Abbreviations

The following abbreviations are used in this manuscript:

ACL	Anterior cruciate ligament
ACLR	Anterior cruciate ligament reconstruction
RTS-TB	Return to sport test battery
PROMs	Patient-reported outcome measures
PRISMA	Preferred reporting items for systematic reviews and meta-analysis
PERSiST	PRISMA in exercise, rehabilitation, sport medicine and sports science
LSI	Limb symmetry index
JBI	Joanna Briggs Institute
RoB	Risk of bias
SWiM	Synthesis without meta-analysis
GRADE	Grading of recommendations assessment, development and evaluation
BAME	Black, Asian and minorities with ethnic backgrounds
CI	Confidence intervals
LET	Lateral extra-articular tenodesis
ICC	Intraclass correlation coefficient
