# Supplementary material for: Pass Rates of Return to Sport Test Batteries Following Anterior Cruciate Ligament Reconstruction: A Systematic Review and Meta-Analysis
Source: Sports (Basel). 2026 May 20;14(5):211. doi: 10.3390/sports14050211 (PMC13211181; doi:10.3390/sports14050211)
Supplement: Supplementary file 1 [file sports-14-00211-s001.zip › PROSPERO protocol.pdf]

PROSPERO protocol — Methods

Anticipated start–end dates
Start: 01 January 2016 (coverage of evidence) • End (search coverage): 22 December 2024
(Registration date will be set by PROSPERO; the conduct of the review should be prospective to registration.)

Review question
Among individuals following **primary anterior cruciate ligament reconstruction (ACL-R)**, what is the **prevalence (pass rate)** on **return-to-sport test batteries (RTS-TB)** that **include quadriceps testing and more than two hop tests**, when the RTS-TB outcome is reported dichotomously as **pass/fail**?

Objective(s)

1) To estimate pooled pass rates on eligible RTS-TBs after primary ACL-R

2) To examine whether timing of testing is associated with pass rates.

Eligibility criteria

Study designs
Observational studies eligible for JBI appraisal tools (e.g., cross-sectional, cohort).
Exclude: case studies, systematic reviews, clinical commentaries.

Participants
Humans following **primary ACL-R**; studies with **exclusively paediatric populations (≤16 years)** excluded.

Interventions/Index tests (Exposure)
RTS-TB that **must** include: (i) **quadriceps testing** and (ii) **>2 hop tests**.

Comparators
Not required (single-group prevalence).

Outcomes
Primary: **RTS-TB pass rate** reported as a **single dichotomous outcome (pass/fail)**.
Secondary (for contextual/meta-regression only): **timing** of RTS-TB administration (post-operative month).

Setting
Any.

Time frame
Publications **01 Jan 2016–22 Dec 2024** (chosen to capture post-guideline practice changes following Wright et al. [reference to be added]).

Language
English-language full text only.

Other exclusions
Studies reporting (1) **≥1 functional test OR ≥2 PROMs subjective tests only** (i.e., without meeting the required RTS-TB composition), (2) **≥8 separate clinical tests** as the construct (outside defined RTS-TB scope), (3) duplicate/overlapping cohorts (retain one; others narratively summarised).

Information sources

Electronic databases: **AMED, CINAHL, MEDLINE, SPORTDiscus (EBSCO), PubMed**.
Additional: **Citation searching** of all studies included at full-text stage.

Dates searched

Comprehensive search run **22 Dec 2024**.

Limits/filters

Peer-reviewed journals; exclude systematic reviews.

Reference management

Covidence (screening/data extraction management) and **EndNote 3** (de-duplication & records).

Search strategy

Search terms developed in **EBSCO** syntax using **PICO-informed keywords**, adapted (not modified) for PubMed.

- Concepts combined with **AND**; synonyms within concepts combined with **OR**.
- Field limits: **Title/Abstract**.
- Full strategies to be provided in **Appendix** (Table of terms and exact strings per database).

Two reviewers conducted the initial database search; **citation searching** completed by one reviewer.

Study selection

- **Screening tool**: Covidence.
- **Process**: Two reviewers will **independently** screen titles/abstracts, then full texts, against prespecified criteria.
- **Disagreements** resolved by discussion; where needed, adjudication by a third team member.
- **Deduplication**: EndNote 3 followed by Covidence import checks.
- **PRISMA** flow diagram to document study selection (PRISMA 2020 checklist framework).

Data extraction

- **Tool**: Covidence data extraction template.
- **Extracted items**: study characteristics (design, setting, country), participant details, ACL-R details if reported, **RTS-TB composition**, definition of **pass/fail**, **numbers passing/total**, **timing** of test administration, notes on overlapping cohorts.
- **Process**: Two reviewers extract independently with consensus resolution.

Risk of bias (individual studies)

- **Tool**: **Joanna Briggs Institute (JBI) critical appraisal tools** for observational designs.
- **Judgement**: Converted to **three-tier classification** (**low / moderate / high**) per study, analogous to Newcastle–Ottawa conceptual levels.
- **Assessors**: Two reviewers independently; consensus resolution.

Data synthesis and statistical methods

Primary synthesis

- Effect measure: **proportion passing RTS-TB** (percentage).
- Meta-analysis: **random-effects proportion meta-analysis** to pool pass rates.
- **Software**: **Stata 18.5 (United Kingdom)**.
- **Overlapping cohorts**: Where overlapping populations are identified, include only the most comprehensive/non-overlapping dataset in quantitative synthesis; report others in **narrative synthesis**.

Heterogeneity

- Assess statistical heterogeneity (e.g., I^2, χ^2). Explore clinical/methodological sources qualitatively (differences in RTS-TB composition, timing).

Meta-regression / subgroup analyses

- **Planned meta-regression**: **Timing** of RTS-TB (months post-op) as a continuous predictor of pass rate.
- (If data permit) sensitivity analyses by **risk of bias tier** and by **RTS-TB composition**.

Small-study effects/publication bias

- If ≥10 studies, assess small-study effects (visual inspection; appropriate tests for proportion meta-analysis if applicable).

Narrative synthesis

- For studies not meta-analysed (e.g., overlapping cohorts), provide structured narrative summary.

Certainty of evidence

- **GRADE** approach tailored to **prevalence estimates** to rate certainty (very low to high) across key domains (risk of bias, inconsistency, indirectness, imprecision, publication bias).

Equity, diversity, and inclusion

- The **three-person** team includes members from **academic, public, and private sectors** and contributors from **under-represented groups** (BAME and female).
- Inclusion aims: participants with ACL injury **regardless of gender, socioeconomic status, country of origin, or sport level**, subject to eligibility criteria.

Reporting and registration

- Reporting will follow the **PRISMA 2020** checklist.
- Any protocol amendments post-registration will be documented with dates and rationale.
