# Supplementary material for: Pass Rates of Return to Sport Test Batteries Following Anterior Cruciate Ligament Reconstruction: A Systematic Review and Meta-Analysis
Source: Sports (Basel). 2026 May 20;14(5):211. doi: 10.3390/sports14050211 (PMC13211181; doi:10.3390/sports14050211)
Supplement: Supplementary file 1 [file sports-14-00211-s001.zip › Supp.1-5.pdf]

**Supplemental material S1.** Full electronic search strategies

**Databases searched:**

AMED

CINAHL

MEDLINE

SportDiscus

PubMed

**Search terms:**

TI ( "anterior cruciate ligament" OR acl ) AND TI ( surgery OR operation OR surgical procedure OR reconstruction OR injury OR graft OR re-rupture OR reinjury OR re-injury OR second ) AND TI ( “reinjury risk” OR “Limb symmetry” OR “return to sport" OR "return to play" ) AND TI ( hop* OR jump* OR strength or function* OR performance OR discharge OR decision OR prevent* OR clinical OR "Return-to-sport" OR "return-to-play" or ability OR test* OR criteria OR battery OR result OR rule OR assessment OR outcome OR asymmetrical* OR “Limb symmetry” )

**Limiters**:

Publication Year: 2016

Publication Date: 20100101

Peer Reviewed

Research Article

**Expanders** - Apply equivalent subjects

**Search modes** - SmartText Searching

**Supplemental material S2.** Table of data extraction

| Table Summary of data extraction | |
|---|---|
| **Study** | Reference, year, country of origin, study design |
| **Population** | Age, sex |
| **Exposure** | Return to sport test batter composition: *strength tests, hop tests, PROMs & functional performance tests* |
| **Outcome** | Return to sport test battery pass rate (%) |

**Supplemental material S3.** Full risk of bias assessments; JBI checklist for prevalence studies

| | 1 | 2 | 3 | 4 | 5 | 6 | 7 | 8 | 9 | |
|---|---|---|---|---|---|---|---|---|---|---|
| **Beischer et al.** | Y | Y | Y | Y | P | P | Y | Y | U | Mod |
| **Broman et al.** | Y | Y | Y | Y | U | U | Y | Y | N | Mod |
| **Edwards et al.** | U | U | Y | Y | N | Y | Y | Y | N | Mod |
| **Grindem et al.** | Y | Y | Y | Y | N | Y | N | Y | Y | Low |
| **Kyritis et al.** | Y | Y | Y | Y | U | U | Y | Y | U | Mod |
| **Losciale et al.** | U | U | Y | Y | U | U | Y | Y | U | Mod |
| **Nawasreh et al.** | U | U | Y | Y | N | N | Y | Y | U | Mod |
| **Paterno et al.** | U | U | Y | Y | N | N | Y | Y | U | Mod |
| **Sousa et al.** | U | Y | Y | Y | U | U | Y | Y | N | Mod |
| **Toole et al.** | U | U | Y | Y | N | U | Y | Y | U | Mod |
| **Van Melick et al.** | U | Y | Y | Y | N | N | Y | Y | P | Mod |
| **Wellsandt et al.** | P | P | P | Y | N | P | Y | Y | N | High |

1. Was the sample frame appropriate to address the target population?
2. Were study participants sampled in an appropriate way?
3. Was the sample size adequate?
4. Were the study subjects and the setting described in detail?
5. Was the data analysis conducted with sufficient coverage of the identified sample?
6. Were Valid methods used for the identification of the condition?
7. Was the condition measured in a standard, reliable way for all participants?
8. Was the appropriate statistical analysis?
9. Was the response rate adequate, and if not, was the low response rate managed appropriately?

*Munn Z, Moola S, Lisy K, Riitano D, Tufanaru C. Methodological guidance for systematic reviews of observational epidemiological studies reporting prevalence and incidence data. Int J Evid Based Healthc. 2015;13(3):147–153.*

**Supplemental material S4.** Statistical analysis of results

**Meta-analysis** All studies

| Study | ES | [95% Conf. Interval] | | % Weight |
|---|---|---|---|---|
| Broman et al. 2023 | **0.28** | **0.24** | **0.32** | **14.65** |
| Van Melick et al. 20 | **0.15** | **0.09** | **0.23** | **14.28** |
| Edwards et al. 2018 | **0.15** | **0.10** | **0.23** | **14.32** |
| Nawasreh et al. 2018 | **0.51** | **0.41** | **0.60** | **13.73** |
| Paterno et al. 2021 | **0.26** | **0.20** | **0.34** | **14.28** |
| Kyritis et al. 2016 | **0.73** | **0.66** | **0.80** | **14.28** |
| Sousa et al. 2017 | **0.23** | **0.18** | **0.29** | **14.46** |
| Random pooled ES | **0.33** | **0.19** | **0.47** | **100.00** |

Heterogeneity chi^2 = **215.34** (d.f. = **6**) p = **0.00**
I^2 (variation in ES attributable to heterogeneity) = **97.21%**
Estimate of between-study variance Tau^2 = **0.03**

Test of ES=0 : z= **4.66** p = **0.00**

**Meta-analysis** Non-professional cohort (sensitivity analysis)

| Study | ES | [95% Conf. Interval] | | % Weight |
|---|---|---|---|---|
| Broman et al. 2023 | **0.28** | **0.24** | **0.32** | **18.43** |
| Van Melick et al. 20 | **0.15** | **0.09** | **0.23** | **16.56** |
| Edwards et al. 2018 | **0.15** | **0.10** | **0.23** | **16.75** |
| Nawasreh et al. 2018 | **0.51** | **0.41** | **0.60** | **14.28** |
| Paterno et al. 2021 | **0.26** | **0.20** | **0.34** | **16.57** |
| Sousa et al. 2017 | **0.23** | **0.18** | **0.29** | **17.41** |
| Random pooled ES | **0.26** | **0.18** | **0.33** | **100.00** |

Heterogeneity chi^2 = **45.10** (d.f. = **5**) p = **0.00**
I^2 (variation in ES attributable to heterogeneity) = **88.91%**
Estimate of between-study variance Tau^2 = **0.01**

Test of ES=0 : z= **6.76** p = **0.00**

**Leave-one-out analysis:** All studies

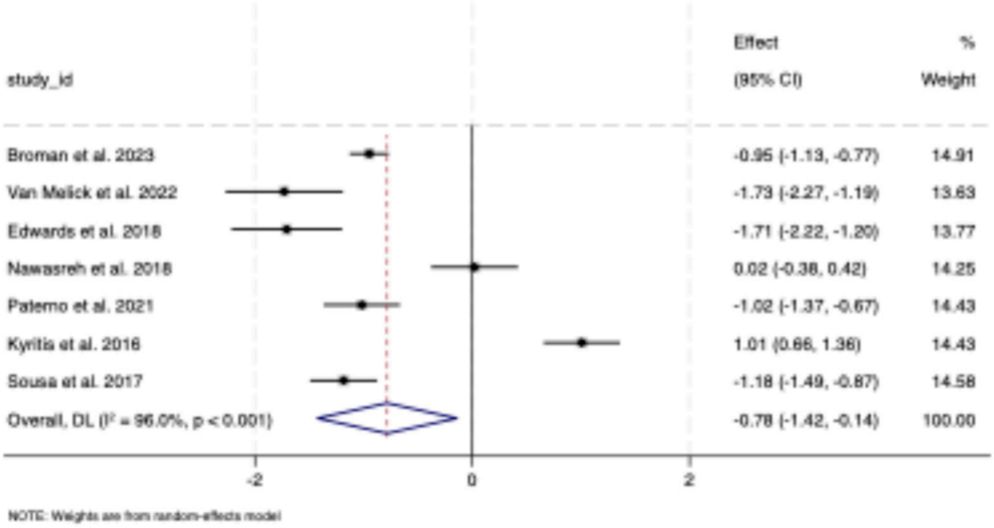


**Leave-one-out analysis:** Non-professional cohort

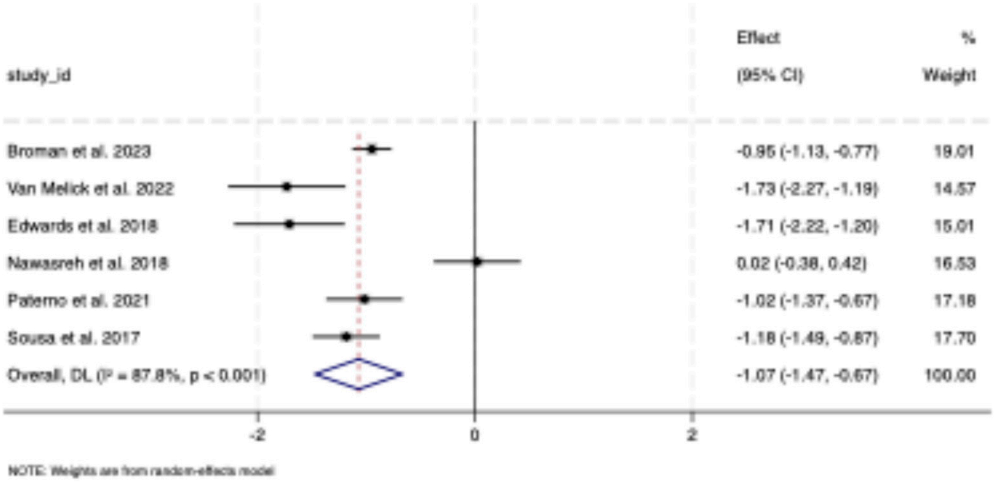

**Meta-regression** All studies

```
Meta-regression                                       Number of obs  =
>  7
REML estimate of between-study variance               tau2           =   .82
> 78
% residual variation due to heterogeneity             I-squared_res  =  99.8
> 7%
Proportion of between-study variance explained        Adj R-squared  =  14.8
> 8%
With Knapp-Hartung modification
-------------------------------------------------------------------------------
> —
  logit_prop | Coefficient  Std. err.      t    P>|t|     [95% conf. interva
> l]
-------------+-----------------------------------------------------------------
> —
months_pos~p |  -.1945761   .1358238    -1.43   0.211    -.5437222    .15457
> 01
       _cons |   .9911293   1.291987     0.77   0.478    -2.330029    4.3122
> 87
-------------------------------------------------------------------------------
> —
```

**Meta-regression** non-professional cohorts

```
Meta-regression                                       Number of obs  =
>  6
REML estimate of between-study variance               tau2           =   .28
> 54
% residual variation due to heterogeneity             I-squared_res  =  99.5
> 3%
Proportion of between-study variance explained        Adj R-squared  =  30.0
> 9%
With Knapp-Hartung modification
-------------------------------------------------------------------------------
> —
  logit_prop | Coefficient  Std. err.      t    P>|t|     [95% conf. interva
> l]
-------------+-----------------------------------------------------------------
> —
months_pos~p |  -.1445919   .0814014    -1.78   0.150    -.3705985    .08141
> 47
       _cons |   .2611559   .7924998     0.33   0.758    -1.939176    2.4614
> 88
-------------------------------------------------------------------------------
> —

.
.
```

## **Supplemental material S5.** PROSPERO protocol Record — Methods

**Anticipated start–end dates**
Start: 01 January 2016 (coverage of evidence) • End (search coverage): 22 December 2024
(Registration date will be set by PROSPERO; the conduct of the review should be prospective to registration.)

**Review question**
Among individuals following **primary anterior cruciate ligament reconstruction (ACL-R)**, what is the **prevalence (pass rate)** on **return-to-sport test batteries (RTS-TB)** that **include quadriceps testing and more than two hop tests**, when the RTS-TB outcome is reported dichotomously as **pass/fail**?

**Objective(s)**

1) To estimate pooled pass rates on eligible RTS-TBs after primary ACL-R

2) To examine whether timing of testing is associated with pass rates.

**Eligibility criteria**

**Study designs**
Observational studies eligible for JBI appraisal tools (e.g., cross-sectional, cohort).
**Exclude**: case studies, systematic reviews, clinical commentaries.

**Participants**
Humans following **primary ACL-R**; studies with **exclusively paediatric populations (≤16 years)** excluded.

**Interventions/Index tests (Exposure)**
RTS-TB that **must** include: (i) **quadriceps testing** and (ii) **>2 hop tests**.

**Comparators**
Not required (single-group prevalence).

**Outcomes**
Primary: **RTS-TB pass rate** reported as a **single dichotomous outcome (pass/fail)**.
Secondary (for contextual/meta-regression only): **timing** of RTS-TB administration (post-operative month).

**Setting**
Any.

**Time frame**
Publications **01 Jan 2016–22 Dec 2024** (chosen to capture post-guideline practice changes following Wright et al. [reference to be added]).

**Language**
**English-language** full text only.

**Other exclusions**
Studies reporting (1) **≥1 functional test OR ≥2 PROMs subjective tests only** (i.e., without meeting the required RTS-TB composition), (2) **≥8 separate clinical tests** as the construct (outside defined RTS-TB scope), (3) duplicate/overlapping cohorts (retain one; others narratively summarised).

**Information sources**

Electronic databases: **AMED, CINAHL, MEDLINE, SPORTDiscus (EBSCO), PubMed**.
Additional: **Citation searching** of all studies included at full-text stage.

**Dates searched**
Comprehensive search run **22 Dec 2024**.

**Limits/filters**
Peer-reviewed journals; exclude systematic reviews.

**Reference management**
**Covidence** (screening/data extraction management) and **EndNote 3** (de-duplication & records).

**Search strategy**

Search terms developed in **EBSCO** syntax using **PICO-informed keywords**, adapted (not modified) for PubMed.

- Concepts combined with **AND** synonyms within concepts combined with **OR**.
- Field limits: **Title/Abstract**.
- Full strategies to be provided in **Appendix** (Table of terms and exact strings per database).

Two reviewers conducted the initial database search: **citation searching** completed by one reviewer.

**Study selection**

- **Screening tool**: Covidence.
- **Process**: Two reviewers will **independently** screen titles/abstracts, then full texts, against prespecified criteria.
- **Disagreements** resolved by discussion; where needed, adjudication by a third team member.
- **Deduplication**: EndNote 3 followed by Covidence import checks.
- **PRISMA** flow diagram to document study selection (PRISMA 2020 checklist framework).

**Data extraction**

- **Tool**: Covidence data extraction template.
- **Extracted items**: study characteristics (design, setting, country), participant details, ACL-R details if reported, **RTS-TB composition**, definition of **pass/fail**, **numbers passing/total**, **timing** of test administration, notes on overlapping cohorts.
- **Process**: Two reviewers extract independently with consensus resolution.

**Risk of bias (individual studies)**

- **Tool**: **Joanna Briggs Institute (JBI) critical appraisal tools** for observational designs.
- **Judgement**: Converted to **three-tier classification** (**low / moderate / high**) per study, analogous to Newcastle–Ottawa conceptual levels.
- **Assessors**: Two reviewers independently; consensus resolution.

**Data synthesis and statistical methods**

**Primary synthesis**

- Effect measure: **proportion passing RTS-TB** (percentage).
- Meta-analysis: **random-effects proportion meta-analysis** to pool pass rates.
- **Software**: **Stata 18.5 (United Kingdom)**.
- **Overlapping cohorts**: Where overlapping populations are identified, include only the most comprehensive/non-overlapping dataset in quantitative synthesis; report others in **narrative synthesis**.

**Heterogeneity**

- Assess statistical heterogeneity (e.g., $\mathbf{I^2}$, $\chi^2$). Explore clinical/methodological sources qualitatively (differences in RTS-TB composition, timing).

**Meta-regression / subgroup analyses**

- **Planned meta-regression**: **Timing** of RTS-TB (months post-op) as a continuous predictor of pass rate.
- (If data permit) sensitivity analyses by **risk of bias tier** and by **RTS-TB composition**.

**Small-study effects/publication bias**

- If ≥10 studies, assess small-study effects (visual inspection; appropriate tests for proportion meta-analysis if applicable).

**Narrative synthesis**

- For studies not meta-analysed (e.g., overlapping cohorts), provide structured narrative summary.

**Certainty of evidence**

- **GRADE** approach tailored to **prevalence estimates** to rate certainty (very low to high) across key domains (risk of bias, inconsistency, indirectness, imprecision, publication bias).

**Equity, diversity, and inclusion**

- The **three-person** team includes members from **academic, public, and private sectors** and contributors from **under-represented groups** (BAME and female).
- Inclusion aims participants with ACL injury **regardless of gender, socioeconomic status, country of origin, or sport level**, subject to eligibility criteria.

**Reporting and registration**

- Reporting will follow the **PRISMA 2020** checklist.
- Any protocol amendments post-registration will be documented with dates and rationale.
